# Light quality rewires module–trait networks in *Euglena gracilis* to drive carotenoid and paramylon accumulation

**DOI:** 10.1186/s13068-026-02774-2

**Published:** 2026-05-29

**Authors:** Ming Du, Zixi Chen, Qingqing Guo, Jing Liu, Ke Xu, Tianming Chen, Chao Li, Yanling Li, Jiangxin Wang

**Affiliations:** 1 Institute for Ecological Research and Pollution Control of Plateau Lakes, School of Ecology and Environmental Science, Yunnan University, Kunming, 650500 Yunnan China; 2 Shenzhen Key Laboratory of Marine Bioresource and Eco-Environmental Science, Shenzhen Engineering Laboratory for Marine Algal Biotechnology, Guangdong Provincial Key Laboratory for Plant Epigenetics, College of Life Sciences and Oceanography, Shenzhen University, Shenzh en, 518060 China; 3 Shenzhen Bay Laboratory, Shenzhen, 518055 China

**Keywords:** Light quality, *Euglena gracilis*, WGCNA, Module–trait association, Carotenoids, Paramylon, Photochemistry (*Fv*/*Fm*), Microalgal bioproducts

## Abstract

**Background:**

Light quality is a critical and tractable parameter for directing carbon partitioning in microalgae toward the synthesis of high-value bioproducts. In this study, *Euglena gracilis* was employed as a model photosynthetic system to investigate spectral regulation of bioproduct accumulation. An integrative approach combining physiological measurements, quantification of major bioproducts (including chlorophylls, carotenoids, lipids, protein, and the β-1,3-glucan paramylon), and RNA-sequencing-based weighted gene co-expression network analysis (WGCNA) was used to dissect spectrum-specific regulatory programs.

**Results:**

Distinct spectral treatments induced divergent photochemical responses (*Fv*/*Fm*) and bioproduct profiles. WGCNA identified 12 transcriptional modules, which were correlated with specific traits and treatments using module eigengene analysis. One module, enriched in carotenoid biosynthesis genes, showed a strong positive correlation with total carotenoid content (*r* ≈ 0.85, *p* < 0.001). A separate module associated with photosystem function and photoprotection closely tracked changes in *Fv*/*Fm* (*r* ≈ 0.75, *p* ≈ 0.03). Notably, a third module responsive to blue-light-dominant conditions displayed positive correlations with chlorophyll a, carotenoids, and paramylon (|*r*| ≈ 0.74–0.79; *p* < 0.05), suggesting an integrated regulatory axis linking light perception, photosynthetic performance, and carbon storage. Additional modules correlated with protein accumulation and broad-spectrum (white) light exposure, implicating coordinated regulation of translational capacity and cytoskeletal dynamics.

**Conclusions:**

Collectively, these module–trait associations elucidate how light quality orchestrates plastidial function, redox homeostasis, isoprenoid biosynthesis, and *β*-1,3-glucan metabolism to reprogram bioproduct yields. This network framework identifies candidate pathway regulators and provides strategic insights for optimizing spectral conditions to enhance carotenoid and paramylon production in *E. gracilis*, supporting advanced microalgal biorefinery development.

**Supplementary Information:**

The online version contains supplementary material available at 10.1186/s13068-026-02774-2.

## Background

Light functions not only as the primary energy source for photoautotrophic microalgae [1] but also as a critical signaling factor that modulates cellular metabolism through photoreceptor-mediated gene regulation and enzymatic control [2, 3]. Because it simultaneously governs energy capture and regulatory networks, light is a central variable in optimizing biomass production and directing carbon flux toward target metabolites. The light environment is typically characterized by intensity and photoperiod, but spectral composition—or light quality—is equally crucial. Variations in light spectrum can influence excitation-energy distribution between photosystems, cellular redox balance, reactive oxygen species (ROS) levels, and subsequent carbon partitioning. Blue and red light, which coincide with chlorophyll absorption peaks, are frequently employed in controlled cultivation systems, such as vertical farms [4, 5] and algal photobioreactors [6–8], with programmable LEDs offering precise and flexible spectral control.

Microalgae are increasingly recognized as sustainable platforms for producing both low-carbon fuels and high-value bioproducts due to their ability to fix CO_2_ and generate energy-rich compounds without relying on arable land or freshwater resources [9, 10]. Nonetheless, large-scale application depends on improving productivity per unit area and ensuring that fixed carbon is efficiently directed into economically relevant product pools. Spectral modulation offers a non-invasive, rapidly adjustable process variable that can be fine-tuned in real time, particularly in LED-equipped cultivation systems. Light quality thus becomes a powerful tool for optimizing production in semicontinuous and indoor photobioreactor operations, where natural irradiance is decoupled from the growth environment.

Among commercially relevant species, *Euglena gracilis* presents a unique opportunity for integrated bioproduct generation. It is metabolically versatile—capable of photoautotrophic, mixotrophic, and heterotrophic growth—and produces several high-value compounds, including the storage polysaccharide paramylon (*β*-1,3-glucan), lipids, vitamins, and diverse bioactive molecules [11, 12]. The emergence of molecular resources such as transcriptomic datasets and draft genome assemblies has further enabled exploration of gene regulatory mechanisms that underlie metabolite synthesis and stress responses [13, 14]. Light-mediated regulation can thus enhance yields of key outputs—pigments, proteins, glucans, and lipid-derived fuels—in algal biotechnology, with implications for future strain engineering and process optimization in biofuels and bioproducts.

The perception and response to light in microalgae involve diverse photoreceptor families that link environmental light cues to physiological and behavioral outcomes [15–17]. In *E. gracilis*, light sensing is mediated in part by the paraflagellar body, a photoreceptive organelle implicated in phototaxis. Biochemical and spectroscopic studies have identified rhodopsin-like molecules and other photoactive proteins as components of the light-sensing machinery [18, 19]. In addition, blue-light responses have been attributed to flavin- and riboflavin-binding proteins localized near the flagellar base, suggesting a complex network of photoreception that modulates motility, cell positioning, and acclimation behavior [20]. These signaling pathways ultimately shape photosynthetic efficiency and mediate trade-offs between growth promotion and photoprotection, making them relevant targets for optimizing spectral conditions in industrial cultivation.

Photosynthetic pigments, particularly chlorophylls and carotenoids, play essential roles in light absorption and photoprotection, allowing microalgae to effectively utilize energy across the photosynthetically active radiation (PAR) spectrum [21]. Carotenoids such as β-carotene and pigments involved in the xanthophyll cycle mitigate excess excitation energy and reduce ROS accumulation, thereby stabilizing photosynthesis under high-light or variable light conditions [22–24]. In *Euglena gracilis*, acclimation to high-light environments involves not only alterations in pigment profiles but also changes in excitation-energy dissipation processes, indicating that photoprotection operates through mechanisms beyond pigment abundance alone [25]. Experimental studies have shown that β-carotene accumulation under high light contributes to photoprotection and acclimation capacity in *E. gracilis* [26]. Furthermore, targeted suppression of phytoene synthase (*EgcrtB*) reduces carotenoid levels and induces intracellular structural changes, further supporting the role of carotenoids in long-term light adaptation [27]. The combined effects of PAR and UV radiation have also been shown to influence phototaxis and photosynthetic efficiency in *E. gracilis*, suggesting that both light quality and light-induced stress modulate cellular behavior and physiology [28].

In addition to mechanistic studies, cultivation-oriented research has demonstrated that environmental parameters interacting with light—such as temperature, CO_2_/O_2_ composition, and irradiance—significantly affect growth and carbon allocation in *E. gracilis* [29]. These findings emphasize that spectral responses must be evaluated within the context of overall cultivation design. Prior engineering studies have already addressed photosynthetic quantum yield and the interactive effects of light quality and CO_2_ supply in *E. gracilis* [30, 31]. Notably, light quality influences not only pigment biosynthesis but also the accumulation of storage compounds. Comparative analyses across *Euglena* strains grown under different light regimes have shown distinct paramylon accumulation patterns, highlighting the potential to steer *β*-1,3-glucan synthesis via spectral optimization [32].

The impact of light quality varies across microalgal taxa. In *Chlorella vulgaris*, maximal biomass has been reported under white or blue light [33], and warm white light has sometimes outperformed red–blue combinations in promoting growth [34]. In *Dunaliella salina*, carotenoid accumulation has been linked to specific spectral conditions, with red light associated with enhanced pigment synthesis under certain circumstances [35–39]. These studies collectively show that optimal light spectra are species- and product-specific, while pigment-level shifts alone do not reliably predict carbon allocation to storage carbohydrates or lipids.

Although light quality clearly influences pigment composition, storage carbohydrate accumulation, and growth, the underlying regulatory mechanisms span processes from light perception and photoprotection to isoprenoid biosynthesis and glucan assembly. This complexity undermines single-gene analyses of spectral effects and reveals a critical gap: previous studies have largely described these responses at the physiological or single-gene level, leaving the higher-order regulatory architecture that coordinates metabolic shifts under different light qualities unexplored. Weighted gene co-expression network analysis (WGCNA) bridges this gap by providing a system-level framework that clusters genes into co-expression modules and correlates their expression with phenotypic traits, enabling identification of pathway-level signatures and regulatory hubs [40, 41]. It constructs robust biological networks using soft-thresholding and topological overlap measures [40], supported by widely used tools for module detection and module–trait correlation [41, 42].

Uncovering the modular organization of transcriptional programs—and the hub genes that drive them—shifts understanding from descriptive phenotypic observations to mechanistic insight into the regulatory logic governing carbon partitioning. Because light quality perturbs multiple interconnected pathways, this network-level perspective offers a more rational basis for strain engineering and spectral optimization than incremental single-gene analyses. Already a standard tool in plant and algal transcriptomics for dissecting pigment metabolism, photosynthesis, and stress responses [43, 44], WGCNA applied to *E. gracilis* under defined light spectra can reveal how spectral cues orchestrate coordinated changes in photosynthetic efficiency, pigment biosynthesis, and carbon allocation toward lipids and paramylon—traits directly linked to the feasibility of bioproduct and biofuel manufacturing.

The present study investigates how specific light wavelengths—particularly blue and red—affect growth rate, photosynthetic performance, and the accumulation of paramylon, total lipids, and proteins in *E. gracilis* [45]. In parallel, we integrate transcriptomic profiling with WGCNA to construct gene co-expression networks and map them onto key phenotypic traits such as *Fv*/*Fm*, pigment levels, and storage compound yields under distinct spectral conditions [40, 41, 43]. This integrative approach identifies candidate regulatory modules and hub genes, offering actionable insights for optimizing spectral inputs and guiding future metabolic engineering efforts aimed at enhancing microalgal bioproductivity.

## Methods

### Strain and culture conditions

*Euglena gracilis* CCAP 1224/5Z was obtained from the Culture Collection of Algae and Protozoa (https://www.ccap.ac.uk/) and maintained at Shenzhen University. Cultivation was carried out in CM medium [46], consisting of: 1 g/L (NH_4_)_2_HPO_4_, 1 g/L KH₂PO₄, 0.2 g/L MgSO_4_·7H_2_O, 0.8 g/L Na_3_C_6_H_5_O_7_·2H_2_O, 0.02 g/L CaCl_2_, 3 mg/L Fe_2_(SO_4_)_3_·nH_2_O, 1.8 mg/L MnCl_2_·4H_2_O, 1.5 mg/L CoSO_4_·7H_2_O, 0.4 mg/L ZnSO_4_·7H_2_O, 0.2 mg/L Na_2_MoO_4_·2H_2_O, 0.02 mg/L CuSO_4_·5H_2_O, 2.48 mg/L H_3_BO_3_, 0.5 mg/L vitamin B₁, and 0.02 g/L vitamin B₁₂. *Euglena gracilis* was pre-cultured for 4 days under different light qualities at 25 °C to reach the exponential growth phase, then used for subsequent experiments. Cultures were maintained at 25 °C under a 12 h light/12 h dark photoperiod with white (450–760 nm), red (635 nm), or blue (453 nm) LED light (40 μmol photons·m^−2^·s^−1^), and the spectra are shown in Fig. S1. Aeration was provided by continuous bubbling with either ambient air (0.04% CO_2_) or 5% CO_2_ (v/v) at a flow rate of 500 mL min⁻^1^. All treatments were conducted in biological triplicates.

### Growth measurement

Cell growth was monitored daily by measuring optical density at 750 nm (OD_750_) using a digital spectrophotometer (ND2000, ThermoFisher). Cultures were inoculated at an initial OD_750_ of 0.1, corresponding to approximately 1 × 10^5^ cells mL⁻^1^. Measurements continued until cultures reached stationary phase.

### Morphological analysis

Cell morphology was assessed by fixing 10 μL of culture with Lugol’s iodine and imaging under an inverted microscope (DMi1, Leica, Germany). Width and length of individual cells were measured using ImageJ software, and the width-to-length ratio was calculated. Cells were categorized morphologically as follows: elongated (0.01–0.30), spindle-shaped (0.31–0.70), or spherical (0.71–1.00), based on established criteria [47, 48].

### Pigment quantification and photosynthetic efficiency (*Fv*/*Fm*)

Chlorophyll and carotenoid contents were determined from 100 mg of freeze-dried *E. gracilis* biomass extracted with 95% ethanol at 4 °C. Absorbance was measured at 470, 649, and 665 nm using a spectrophotometer (ND2000, ThermoFisher). Pigment concentrations were calculated using the following standard equations [49]:

$${\mathrm{Chlorophyll}}\;{\mathrm{a}}\;\left( {{\mathrm{mg}}/{\mathrm{L}}} \right) = {13}.{95} \times A_{655} - {6}.{88} \times A_{649}$$

$${\mathrm{Chlorophyll}}\;{\mathrm{b}}\;\left( {{\mathrm{mg}}/{\mathrm{L}}} \right) = {24}.{96} \times A_{{{649}}} - {7}.{32} \times A_{{{665}}}$$

$${\mathrm{Carotenoids}}\;\left( {{\mathrm{mg}}/{\mathrm{L}}} \right) = ({1}000 \times A_{{{47}0}} - {2}.0{5} \times {\mathrm{Chl}}\;{\mathrm{a}} - {114}.{8} \times {\mathrm{Chl}}\;{\mathrm{b}})/{245}$$

For chlorophyll fluorescence, 3 mL of algal culture was dark-adapted for 15 min prior to measurement. Maximum quantum yield of PSII (*Fv*/*Fm*) was assessed using a pulse-amplitude modulated fluorometer (Image-PAM, WALZ PHYTO-C, Germany) with a quartz cuvette, following standard protocols [50].

### Total lipid content

Total lipids were extracted from 100 mg of freeze-dried algal biomass using a modified Bligh and Dyer protocol [51]. Cells were first sonicated for 30 min in 9.5 mL of a pre-mixed solvent system (chloroform:methanol:water = 1:2:0.8, v/v/v). After centrifugation, the supernatant was collected and phase separation was induced by adjusting the solvent ratio to chloroform:methanol:water = 1:1:0.9. The lower organic phase was isolated by centrifugation, transferred into pre-weighed glass tubes, and dried under nitrogen gas. Lipid content was determined gravimetrically.

### Paramylon quantification

Paramylon content was measured using the protocol described by Takenaka [52], with slight modifications. One hundred milligrams of dried algal biomass were ground and washed with 95% ethanol to remove residual lipids. After centrifugation, the pellet was treated with 15 mL of 1% (w/v) sodium dodecyl sulfate (SDS) at 85 °C for 30 min to solubilize proteins. The suspension was centrifuged and washed twice with deionized water. The remaining paramylon pellet was transferred to a pre-weighed tube, oven-dried at 50 °C, and quantified by dry weight.

### Protein quantification

Total soluble protein was quantified using the bicinchoninic acid (BCA) assay (Kit No. PC0020, Solarbio). Aliquots (20 μL) of cell lysate were pipetted into a 96-well microplate, followed by the addition of 200 μL of BCA working reagent. The plate was incubated at 37 °C for 30 min, and absorbance was measured at 562 nm. Protein concentrations were calculated from a standard curve constructed with bovine serum albumin.

### Transcriptomic analysis

Eighteen samples (six light/CO₂ treatment conditions, each with three biological replicates) were used for transcriptomic analysis. Total RNA was extracted, and mRNA was purified and reverse-transcribed to cDNA. Following library construction, fragments were size-selected and quantified using qRT-PCR. High-throughput sequencing was performed using the Illumina NovaSeq 6000 platform. De novo assembly was conducted using Trinity, and transcriptome quality was assessed as described previously [53]. Coding sequences (CDS) were predicted from assembled unigenes. Differential gene expression analysis was performed using the DESeq2 R package (v1.20.0), and functional annotation was carried out via Gene Ontology (GO) and Kyoto Encyclopedia of Genes and Genomes (KEGG) databases.

### Weighted gene co-expression network analysis (WGCNA)

To identify light-responsive gene networks, WGCNA was applied to the variance-stabilized RNA-seq expression matrix. Genes with low expression (maximum fragments per kilobase million [FPKM] ≤ 1 across all samples) were excluded. Sample and gene quality were verified using hierarchical clustering and the goodSamplesGenes function, with outliers removed based on dendrogram inspection [41].

A signed-hybrid co-expression network was constructed. The soft-thresholding power (*β*) was determined using the pickSoftThreshold function to approximate scale-free topology. A *β* value of 30 was selected based on high scale-free fit (*R*^2^ > 0.8 Fig. S2) and acceptable mean connectivity. The topological overlap matrix (TOM) was calculated, and genes were clustered using TOM-based dissimilarity. Modules were identified via dynamic tree cutting and merged using a threshold of mergeCutHeight = 0.25, resulting in 12 distinct modules (Fig. S3) for downstream analysis [41].

A trait matrix was constructed using binary encodings of spectral treatments (blue, red, white) and continuous measurements of *Fv*/*Fm*, pigment content (chlorophyll a, chlorophyll b, carotenoids), storage compounds (paramylon, total lipids), protein content, and morphology index. Module–trait correlations were computed via Pearson correlation, and statistical significance was assessed using the Student’s t-distribution. Multiple testing correction was performed using the Benjamini–Hochberg method (FDR < 0.05) [54].

Gene significance (GS) and module membership (kME) values were calculated as the absolute correlation between gene expression and phenotypic traits, and between gene expression and module eigengenes, respectively. Hub gene candidates were defined as those with high GS (≥ 0.5) and high kME (≥ 0.7) within modules significantly associated with measured traits [40, 41].

Functional enrichment analyses for trait-associated modules were performed using the GO and KEGG frameworks (FDR-adjusted *p* < 0.05). Network visualizations included sample and gene dendrograms, module–trait heatmaps, and intramodular co-expression subnetworks exported for selected modules [55].

### Statistical analyses

Statistical analyses were performed using GraphPad Prism (v9.0, GraphPad Software, La Jolla, CA, USA). Two-way analysis of variance (ANOVA) was applied to test the effects of light quality and CO₂ concentration, as well as their interaction, on physiological and biochemical parameters, including pigment content, storage compounds, protein, and *Fv*/*Fm*. Where interactions were significant (*p* < 0.05), Tukey’s *post hoc* test was used for multiple comparisons. Differences were indicated using lowercase letters (e.g., a, b, c) in graphical representations. All experiments were conducted with at least three biological replicates, and data are presented as mean ± standard error (SE).

## Results

### Effects of light quality and CO_2_ on growth

**Fig. 1:**
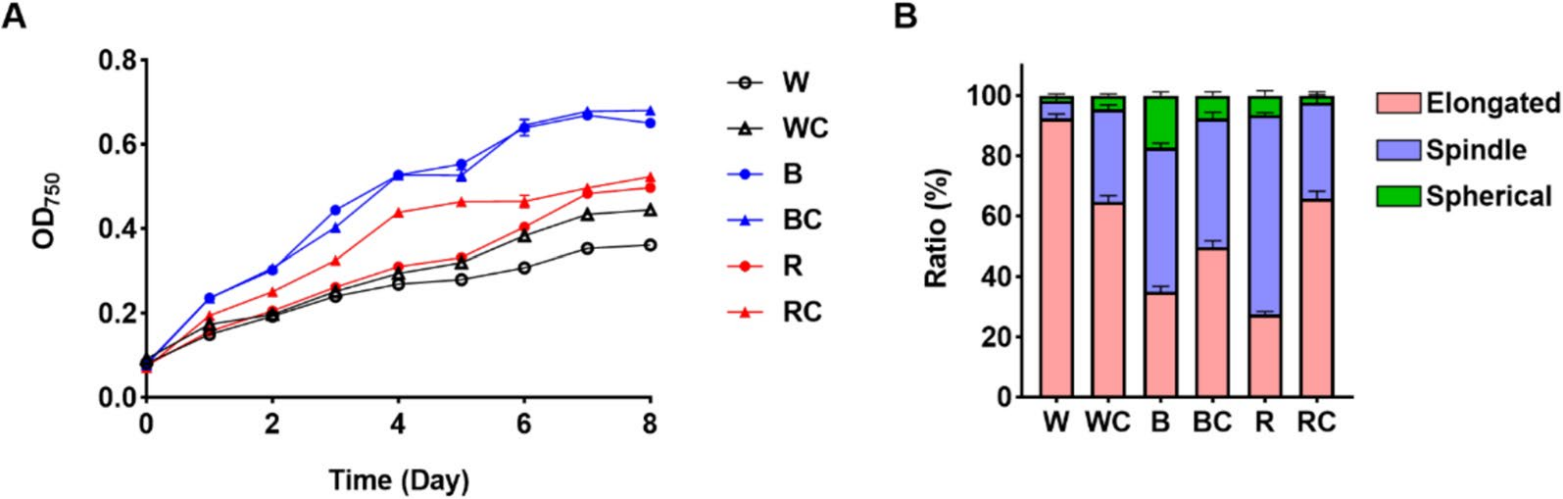
Growth curves and cell morphology under different light qualities. **A** Growth curves of *E. gracilis* under three different light qualities. **B** Morphological alterations of *E. gracilis* during cultivation. W, B, R represent the different light qualities of white, blue, and red, respectively. **C** Ventilation of 5% CO_2_. The data presented represent the mean values with standard deviation (mean ± SE) obtained from three independent replicates (*n* = 3)

*Euglena gracilis* was cultivated under three spectral conditions (blue, red, white) and two CO_2_ levels (ambient and 5%) for 7 days until reaching the stationary phase. Biomass accumulation trends are presented in Fig. 1A. Under elevated CO_2_ (5%), cell growth was significantly accelerated during the exponential phase, particularly under red and white light, and continued to increase through the stationary phase. The most pronounced biomass accumulation, however, was observed under blue light. Specifically, the highest cell densities were achieved under blue light combined with 5% CO_2_ (BC), with increases of 53.4% and 80.0% relative to white light under elevated (WC) and ambient CO_2_ (W), respectively. Red light treatments (RC and R) resulted in biomass concentrations 1.18-fold and 1.37-fold higher than those under WC and W, respectively.

Both light quality and carbon supply had significant effects on *E. gracilis* growth. Among spectral treatments, blue light supported the highest cell densities, followed by red and white light. Across all light conditions, the addition of 5% CO_2_ significantly enhanced biomass accumulation compared to ambient CO_2_. These findings confirm the synergistic impact of high CO_2_ and specific light spectra—particularly blue and red—on promoting algal proliferation.

### Effects on cell morphology

Cell morphology, a key physiological indicator linked to growth and metabolism in *E. gracilis*, varied notably across treatments (Fig. 1B). Three primary morphotypes were observed: elongated, spindle-shaped, and spherical cells. Under ambient CO_2_ and white light, ~ 92% of cells exhibited an elongated morphology, consistent with a default growth state. In contrast, spindle-shaped morphologies predominated under blue and red light conditions, with blue light also inducing a higher proportion of spherical cells (> 16%).

Under elevated CO_2_ (5%), these spectral effects were further accentuated. In particular, blue light combined with 5% CO_2_ (BC) produced the highest proportions of spherical (9%) and spindle-shaped (42%) cells. Under red light with 5% CO_2_ (RC), the ratio of spindle-shaped cells was approximately tenfold higher than under white light.

These morphological shifts are indicative of changes in cellular metabolism and proliferation state. The observed increase in spherical and spindle-shaped forms under blue and red light suggests activation of developmental programs that may relate to cell cycle progression and intracellular resource allocation [56, 57]. Moreover, the interaction with elevated CO_2_ points to coordinated regulation between carbon availability and light-responsive morphology in *E. gracilis*.

### Effects on pigment content and photosynthetic efficiency (*Fv*/*Fm*)

**Fig. 2:**
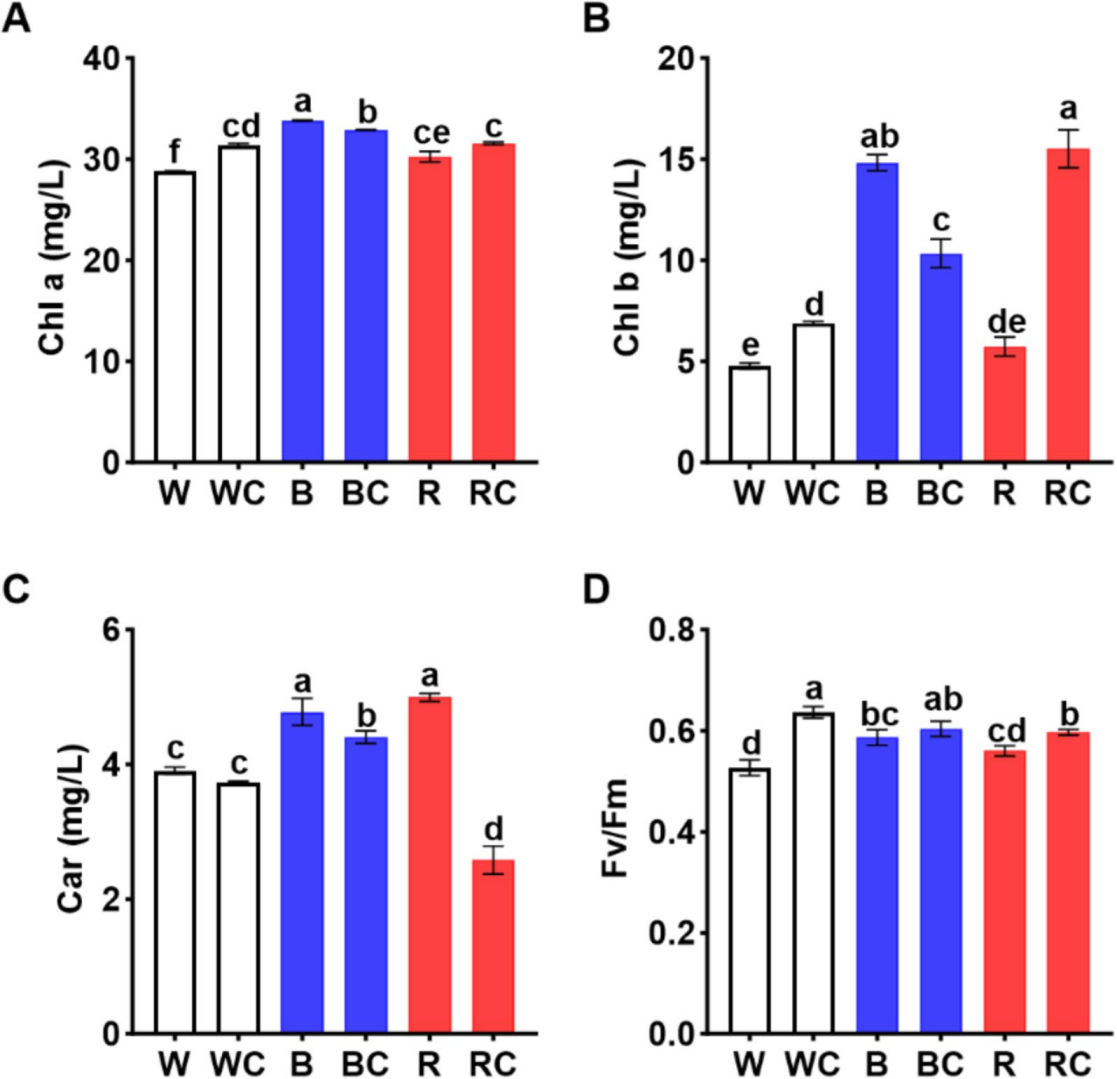
Effects of light qualities on photosynthetic pigments (**A**–**C**), and *Fv*/*Fm* ratio (**D**). Different letters (a, b, c, d, e, f) indicate significant difference according to the ANOVA test at *p* < 0.05. The values represent the mean ± SE, where *n* = 3

*Euglena gracilis* produces chlorophyll a, chlorophyll b, and carotenoids, all of which are integral to light harvesting and photoprotection [58]. Pigment contents under different light qualities and CO_2_ levels are shown in Fig. 2A–C. Blue light significantly promoted pigment accumulation compared with white light. Specifically, under ambient CO_2_, chlorophyll a, chlorophyll b, and carotenoids increased to 33.8 mg L⁻^1^, 14.8 mg L⁻^1^, and 4.76 mg L⁻^1^, respectively. Red light similarly enhanced chlorophyll b and carotenoids, reaching 15.5 mg L⁻^1^ and 5.0 mg L⁻^1^, respectively.

CO_2_ supplementation further stimulated pigment biosynthesis. Under 5% CO_2_, both chlorophyll a and chlorophyll b levels increased relative to ambient CO₂, with blue and red light showing the most pronounced effects. These results confirm that both spectrum and inorganic carbon availability modulate pigment accumulation in *E. gracilis*.

The maximum photochemical efficiency of photosystem II (*Fv*/*Fm*), a proxy for photosystem integrity and stress response, is presented in Fig. 2D. Under ambient CO_2_, *Fv*/*Fm* values were 0.59 for blue light and 0.53 for white light. Under elevated CO_2_ (5%), *Fv*/*Fm* increased across all treatments, with the most notable improvements under red and white light. No significant *Fv*/*Fm* differences were observed among light qualities under 5% CO_2_, suggesting a CO_2_-mediated stabilization of photochemistry.

### Effects on biochemical composition

**Fig. 3:**
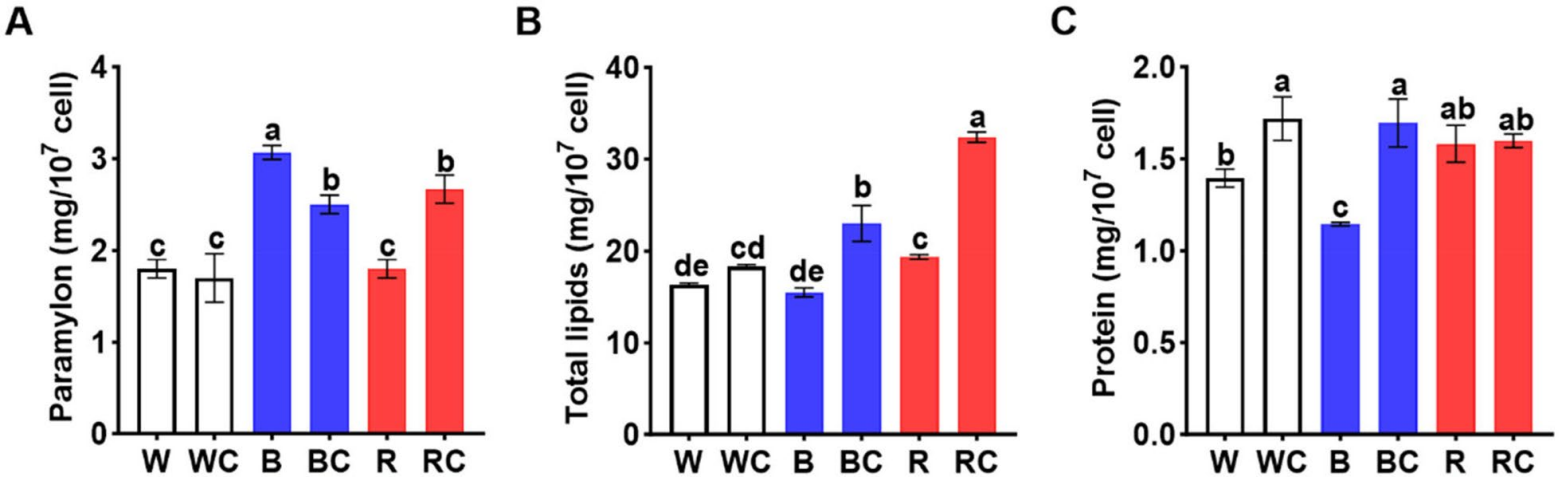
Effects of light qualities on lipids (**A**), paramylon (**B**), and protein (**C**) content. Different letters (a, b, c, d, e) indicate significant difference according to the ANOVA test at *p* < 0.05. The values represent mean ± SE, where *n* = 3

The biochemical profiles of *E. gracilis*—paramylon, total lipids, and protein—are summarized in Fig. 3A–C. Lipid accumulation was markedly enhanced under 5% CO_2_. The highest lipid content was observed under red light with 5% CO_2_, reaching 32.4 ± 0.59 mg per 10^7^ cells, a 77.0% increase compared to white light with 5% CO_2_ (18.3 mg per 10^7^ cells). Under blue light with 5% CO_2_, lipid content reached 23.0 mg per 10^7^ cells, representing a 25.7% increase over white light.

Paramylon, the *β*-1,3-glucan storage polysaccharide, showed strong accumulation under blue and red light (Fig. 3B). The highest value occurred under blue light with ambient CO_2_ (B), reaching 3.07 mg per 10^7^ cells—a 70.6% increase relative to white light. Red light with 5% CO_2_ also significantly enhanced paramylon to 2.67 mg per 10^7^ cells, a 57.1% increase over white light. These data suggest that spectral quality, particularly blue light, has a pronounced effect on directing fixed carbon into storage glucans.

Protein content, presented in Fig. 3C, exhibited smaller but statistically relevant shifts. Under 5% CO_2_, protein content increased by 23.7% and 48.2% under white and blue light, respectively, compared to ambient CO_2_. However, protein levels under blue light were 21.9% lower than under white light, indicating that blue-light exposure may favor pigment and paramylon synthesis over protein accumulation. This suggests a reallocation of carbon resources toward secondary metabolite pools under specific light–CO_2_ combinations.

### RNA-seq overview and differential gene expression

Transcriptomic analysis was conducted to uncover regulatory mechanisms underlying the observed physiological responses. Each sample yielded approximately 23.4 million raw reads, with ~ 22.3 million clean reads retained post-filtering. Quality metrics were high, with Q20 and Q30 values of 97% and 92%, respectively. The mean sequencing error rate was ~ 3%.

Differential gene expression analysis was performed using white-light conditions as the reference (Table 1). The most extensive transcriptomic changes occurred under red light with 5% CO_2_ (RC), with 4,012 genes up-regulated and 5,520 genes down-regulated. Under ambient CO_2_, blue light-induced substantial changes (878 up-regulated and 1,049 down-regulated genes). Red light under ambient CO_2_ produced comparatively fewer expression changes (130 up-regulated, 213 down-regulated).

**Table 1:** Differentially expressed genes (DEGs) under different treatments

Treatments	Total DEGs	Up-regulated	Down-regulated
W vs. WC	1074	777	297
B vs. BC	1273	447	826
R vs. RC	9532	5520	4012
B vs. W	1927	878	1049
R vs. W	343	130	213
BC vs. WC	1870	1288	582
RC vs. WC	1667	752	915

Treatments include exposure to blue light (B), red light (R), and white light (W). C represents treatment with 5% CO_2_

These results align with the physiological data, suggesting that blue and red light, particularly when combined with elevated CO_2_, activate large-scale transcriptional reprogramming that drives changes in growth, pigment content, and storage compound accumulation.

### Functional enrichment analysis (GO and KEGG)

Gene Ontology (GO) enrichment analysis revealed distinct transcriptional responses to spectral quality and CO_2_ availability. Under blue light with ambient CO_2_, up-regulated categories included ribosome biogenesis and organelle-associated processes. In contrast, red light under the same CO_2_ condition led to significant down-regulation of genes involved in thylakoid structure, energy precursor generation, and oxidoreductase activity. Under red light with 5% CO_2_, multiple gene categories—including ribosome biogenesis, signal transduction, plasma membrane organization, and photosynthesis—were up-regulated.

Kyoto Encyclopedia of Genes and Genomes (KEGG) pathway analysis further highlighted spectrum- and CO_2_-specific regulation. Blue light with ambient CO_2_ up-regulated genes involved in photosynthesis, ribosome biogenesis, and antigen processing and presentation. In blue light with 5% CO_2_, enriched pathways included PI3K–Akt signaling, FoxO signaling, mTOR signaling, MAPK signaling, RNA transport, and RNA degradation. In red light with 5% CO_2_, photosynthetic pathways were up-regulated relative to ambient CO_2_, whereas PI3K–Akt, mTOR, and RNA transport pathways were down-regulated. These findings indicate spectrum-dependent modulation of both core metabolic and stress-response pathways.

### Expression profiles of photosynthesis-related genes under blue light and elevated CO_2_

**Fig. 4:**
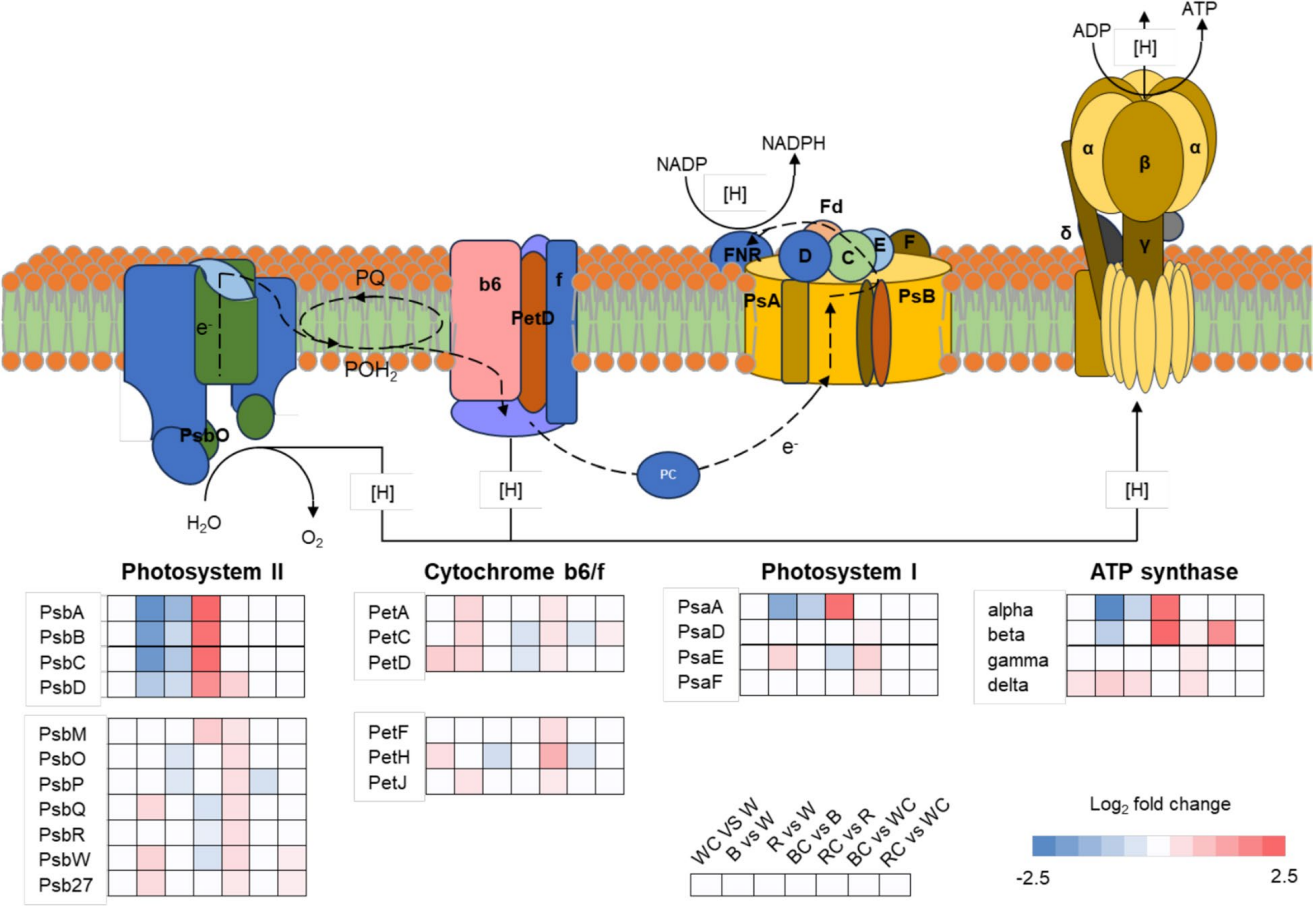
Changes in transcript abundance of the genes involved in photosynthesis of *E. gracilis*

Photosynthetic electron transport involves complexes localized to the thylakoid membrane, including photosystems I and II, the cytochrome b₆/f complex, and ATP synthase [59]. Expression analysis identified 20 genes associated with these components that were differentially regulated (Fig. 4). Under 5% CO_2_, most photosynthetic genes were moderately up-regulated (1.08–1.36-fold) under red and white light compared to ambient CO_2_.

Under blue light, several genes showed pronounced up-regulation: *psbQ*, *psbW*, and *psb27* (PSII), *psaE* (PSI), *petC*, *petD*, *petJ* (cytochrome b₆/f), and *atpδ* (ATP synthase), with fold changes of 1.28–1.44 relative to white light. Conversely, *psbA*, *psbB*, *psbC*, and *psbD* (PSII), as well as *atpα* and *atpβ*, were down-regulated under both blue and red light. These expression patterns suggest enhanced linear electron flow and photochemical activity under blue light with elevated CO_2_, consistent with observed increases in *Fv*/*Fm* and pigment levels.

### Expression of photoreceptor genes under blue and red light

Cryptochromes, which mediate responses to UVA and blue light, play essential roles in DNA repair, pigment biosynthesis, and developmental regulation [60]. As shown in Table 2, cryptochrome-related transcripts were up-regulated by approximately 1.05–1.73-fold under blue light. Notably, deoxyribosylcytosine photolyase was strongly induced (~ 10.33-fold), indicating enhanced light-dependent DNA repair activity.

**Table 2:** Gene expression of *Euglena gracilis* under different lighting qualities

Group	Gene ID	NR	Fold change
B vs. W	Cluster-5366.19719	Cryptochrome	1.21
	Cluster-5366.18686	Cryptochrome	1.14
	Cluster-5366.3999	Cryptochrome	1.05
	Cluster-5366.32462	Deoxyribosylcytosine photolyase	1.73
	Cluster-5366.2187	UVR 3-like DNA photolyase	1.13
BC vs. WC	Cluster-5366.19719	Cryptochrome	1.17
	Cluster-5366.32462	Deoxyribosylcytosine photolyase	10.33
	Cluster-5366.2187	UVR 3-like DNA photolyase	1.02
R vs. W	Cluster-5366.13023	Phytochrome	1.15
RC vs. WC	Cluster-5366.18978	Serine/threonine-protein phosphatases	1.10
	Cluster-5366.21956	Phytochrome	1.07
	Cluster-5366.13023	Phytochrome	1.07

Phytochrome-related transcripts, responsive to red/far-red light, were up-regulated by approximately 1.07–1.15-fold under red light vs. white light [61]. These results support a model in which *E. gracilis* enhances spectral information processing and light stress adaptation via up-regulation of specific photoreceptor gene families under corresponding spectral conditions.

### Regulation of cytoskeletal genes under blue light

Cytoskeletal remodeling, particularly involving actin dynamics, underpins morphological plasticity in response to environmental signals [62]. Under blue light, several cytoskeleton-associated genes exhibited increased expression, including *MEK1*, *PPP1C*, and *PFN* (profilin), with fold changes of 1.35–1.86 compared to white light (Table 3). Serine/threonine phosphatase PPKL1 (EC 3.1.3.16) was also significantly up-regulated. The induction of *PFN* and *PPKL1* suggests that blue light triggers a transcriptional program favoring cytoskeletal rearrangement, which may explain the observed transitions toward spindle-shaped and spherical cells under this condition.

**Table 3:** Changes in transcript abundance of the genes involved in cytoskeleton

Gene ID	NR	Group	Fold change
Cluster-5366.24770	Profilin	B vs. W	1.86
		R vs. RC	0.71
Cluster-5366.14383	Serine/threonine-protein phosphatase PP1 catalytic subunit	B vs. W	1.43
		BC vs. WC	1.35
		R vs. RC	1.19
Cluster-5366.22884	1-Phosphatidylinositol-4-phosphate 5-kinase	B vs. BC	1.38
		B vs. W	0.77
		R vs. W	0.72
		BC vs. WC	0.79
		RC vs. WC	0.84
Cluster-5366.18839	Mitogen-activated protein kinase kinase 1	B vs. BC	0.70
		B vs. W	1.60
		R vs. RC	1.17
		BC vs. WC	1.40
Cluster-5366.27224	Actin beta/gamma 1	BC vs. WC	0.75

### Co-expression network analysis across spectral treatments

**Fig. 5:**
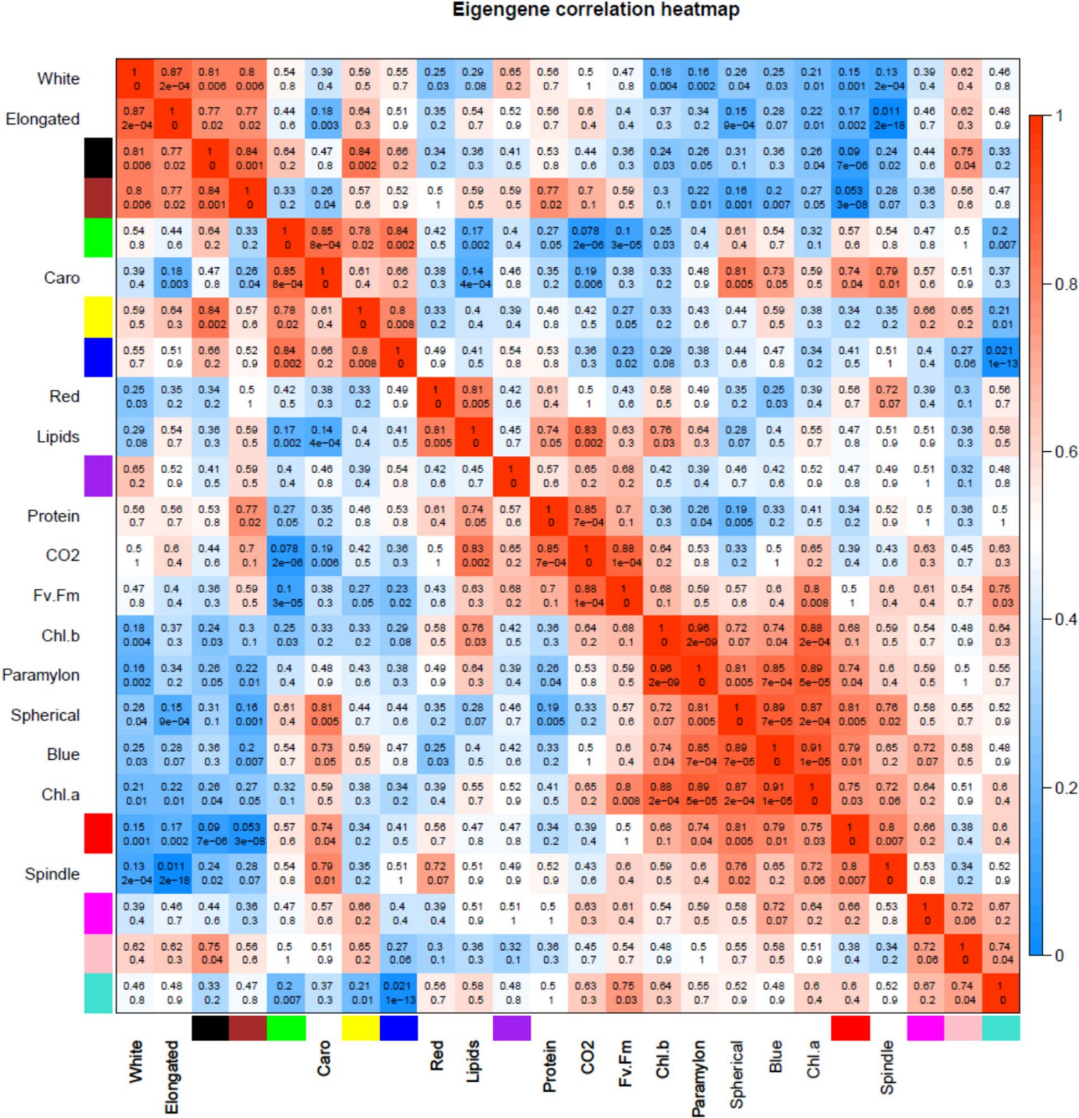
Correlation matrix of experimental treatments, key phenotypic traits and gene expression modules

To identify coordinated transcriptional programs, we performed weighted gene co-expression network analysis (WGCNA) on the normalized RNA-seq data. A signed-hybrid network was constructed using a soft-thresholding power of *β* = 30, which achieved scale-free topology (*R*^2^ > 0.8). Genes were clustered by topological overlap, and modules were defined using dynamic tree cutting and merged at mergeCutHeight = 0.25. This analysis yielded twelve discrete co-expression modules, each assigned a unique color identifier (Fig. 5). Genes not clustered into any module were grouped in the “grey” category and excluded from interpretation.

These modules provide a structured representation of how *E. gracilis* transcriptomes reorganize in response to different light spectra and CO_2_ concentrations, forming the basis for downstream trait association and hub gene analysis (see Sect. "Discussion").

### Module–trait relationships across spectral treatments

To dissect transcriptional coordination underlying spectral responses, module eigengenes derived from WGCNA were correlated with experimental treatments (blue, red, and white light) and key phenotypic traits, including *Fv*/*Fm*, chlorophyll a and b, total carotenoids, paramylon, total lipids, protein, and cell morphology index. The correlation matrix is visualized in Fig. 5, with Pearson correlation coefficients and corresponding *p* values.

Several module–trait associations were statistically significant and biologically informative. The turquoise module showed a moderately strong positive correlation with *Fv*/*Fm* (*r* ≈ 0.75, P ≈ 0.033), consistent with a role in regulating photochemical efficiency. A strong positive correlation was observed between the green module and total carotenoids (*r* ≈ 0.85, *P* = 7.99 × 10⁻^4^), suggesting its control over carotenoid biosynthetic capacity. The red module exhibited positive correlations with blue-light response, chlorophyll a, carotenoids, and paramylon (*r* ≈ 0.74–0.79, *P* < 0.05), indicating a coordinated regulatory axis from light sensing to pigment accumulation and storage β-glucan biosynthesis. The brown module was significantly positively correlated with protein content (*r* ≈ 0.77, *P* ≈ 0.0184), implying its regulation of translation and protein biosynthesis. Furthermore, the black module displayed a positive correlation with the white-light treatment (*r* ≈ 0.81, *P* = 0.0055), suggesting a white-light-specific transcriptomic programming potentially linked to cytoskeletal and growth-related processes.

These module–trait associations are congruent with physiological trends reported in Sects. “Effects of light quality and CO2 on growth”–“Effects on biochemical composition”, including enhanced pigment accumulation under blue light, lipid increases under red light with CO_2_ supplementation, and changes in morphology and protein levels under white light. Together, the WGCNA results support a model in which spectral quality orchestrates a system-level redistribution of carbon among key bioproduct pools.

### Functional enrichment and candidate hub genes in trait-associated modules

Gene ontology and KEGG enrichment analyses of trait-associated modules revealed coherent biological functions that underpin the observed phenotypes. The turquoise module was enriched for terms related to photosystem assembly, photoprotection, and repair pathways, consistent with its correlation with *Fv*/*Fm* and implicating its constituent genes in the maintenance of photosynthetic performance under varied light spectra. Enrichment within the green module highlighted genes involved in isoprenoid biosynthesis and carotenoid modification, directly reflecting its strong association with total carotenoid levels. Functional analysis of the red module showed significant enrichment in chlorophyll biosynthesis, light-harvesting complex organization, and *β*-1,3-glucan (paramylon) metabolic pathways, corroborating the observed co-accumulation of photosynthetic pigments and storage glucans under blue-light conditions. Finally, both the brown and black modules were enriched for components related to the translation machinery, cytoskeletal dynamics, and membrane transport, which align with their respective associations with protein content and morphological changes under white-light exposure.

Within each module, candidate hub genes were identified based on high intramodular connectivity (*kME* ≥ 0.7) and strong trait correlation (gene significance, GS ≥ 0.5). These hub genes represent potential regulatory drivers of spectrum-dependent physiological responses. Representative visualizations of module-specific subnetworks are presented in Supplementary Fig. S1.

These findings nominate functionally relevant gene sets and potential master regulators for future experimental validation and metabolic engineering aimed at optimizing carotenoid, paramylon, and protein production in *E. gracilis* under tailored light regimes.

## Discussion

### Interplay between light quality and CO_2_ availability shapes photochemical efficiency and product allocation

This study highlights the importance of considering light quality and CO₂ availability as interacting variables, rather than independent cultivation parameters. Our findings demonstrate that while CO_2_ enrichment (5%) consistently increased biomass and improved photosynthetic efficiency (*Fv*/*Fm*), the spectral composition of light dictated how assimilated carbon was partitioned into pigments, lipids, and storage carbohydrates. This interplay reinforces the notion that carbon supply modulates the physiological impact of spectral cues—by buffering excitation pressure and maintaining electron transport capacity.

Previous work on *Euglena gracilis* supports this interpretation. Matsumoto et al. reported that under ambient CO_2_ (0.04%), spectral differences had strong effects on growth and nutrient accumulation, whereas at elevated CO_2_ levels (10%), these spectral effects diminished, suggesting that carbon availability becomes the dominant factor when photosynthetic sinks are saturated [31]. In a related study, the quantum yield of CO_2_ fixation in *E. gracilis* peaked under low PPFD and a finely tuned red-to-blue photon flux ratio (9:1), emphasizing the need to co-optimize light spectrum and inorganic carbon delivery for energy-efficient biomass production [30].

The mechanistic basis of this interaction lies in the coupling between light absorption and downstream carbon assimilation. Under CO_2_-limited conditions, reduced carbon fixation capacity leads to accumulation of excess excitation energy, which in turn elevates ROS production and activates photoprotective responses—often at the expense of biomass or product accumulation. By contrast, under CO_2_-replete conditions, the enhanced carbon assimilation provides sufficient sinks for photogenerated electrons, reducing photoinhibition risk and allowing for more stable operation of photosystems. This model is supported by evidence from high-light acclimation studies in *E. gracilis*, which report adjustments in pigment composition—particularly increases in chlorophyll b and xanthophyll-cycle carotenoids—as part of a photoprotective response [25].

In this context, our results underscore that spectrum engineering and CO_2_ enrichment should be treated as synergistic levers rather than separate cultivation strategies. Their combined effect defines the balance between photochemistry and downstream carbon flow, thereby shaping productivity outcomes in biorefinery applications.

### Blue and red light reshape pigment pools and photochemical efficiency

Our findings confirm that *Euglena gracilis* responds to different light spectra through distinct pigment adjustments and photochemical adaptations. Specifically, spectral treatments influenced chlorophyll a/b ratios, carotenoid levels, and *Fv*/*Fm*, suggesting a wavelength-specific acclimation process involving coordinated changes in light harvesting, photoprotection, and electron transport efficiency.

Blue light is particularly noteworthy, as it combines high excitation pressure with strong signaling potential. In *E. gracilis*, blue light induces the biosynthesis of carotenoids, including xanthophyll-cycle pigments, and has been associated with structural modifications, such as thylakoid remodeling and lipid droplet formation [27]. This corresponds well with the “carotenoid-associated” module identified through WGCNA, which exhibited strong correlations with both total carotenoids and *Fv*/*Fm*. The module likely represents a regulatory program involving coordinated expression of isoprenoid biosynthesis genes, activated in response to blue-dominant spectral inputs.

Carotenoids in *Euglena* serve more than photoprotective roles. Tanno et al. demonstrated that pre-irradiation with blue light enhances photosynthetic performance and resilience under subsequent high-light stress, mediated in part by *β*-carotene accumulation [26]. In our data, blue-light treatments correlated with both elevated carotenoid content and improved *Fv*/*Fm*, suggesting that pigment accumulation supports sustained photosynthetic function under spectrally challenging conditions.

It is worth considering whether the observed carotenoid accumulation reflects a specific spectral photo-adaptation rather than a non-specific stress response. Several lines of evidence argue against a general photo-oxidative stress interpretation in our experiments. First, the photon flux density used in all treatments was maintained within the optimal growth range for Euglena gracilis, and the maximum quantum yield of PSII (*Fv*/*Fm*) remained stable at approximately 0.75 across treatments, indicating the absence of chronic photoinhibition. Second, no significant accumulation of reactive oxygen species markers or growth inhibition was detected. Third, and most importantly, our WGCNA and enrichment analyses revealed that transcriptomic responses were not dominated by generic stress-related pathways; instead, the differentially co-expressed gene sets were specifically enriched for carotenoid biosynthesis and light-harvesting complex components. This coordinated regulatory pattern is more consistent with a light-quality-driven photo-adaptive reprogramming of metabolism than with a passive stress response.

Consistent with the interplay described above, the spectral sensitivity of *E. gracilis* decreases under CO_2_-enriched conditions, with blue-light-induced stress effects being attenuated [31]. Thus, optimizing spectral input without adequate CO_2_ supplementation may result in inefficient photochemistry and limited yield.

### Spectral regulation of storage carbon and lipid accumulation through a light–photosynthesis–allocation axis

A central contribution of this study is the identification of a system-level connection between light quality, photosynthetic performance, and downstream carbon allocation. Through WGCNA, modules positively correlated with chlorophyll a, carotenoid content, and paramylon accumulation support a conceptual axis linking spectral signal perception to carbon fixation and eventual partitioning into storage carbohydrates.

This mechanistic framework aligns with prior cultivation studies showing that paramylon yield in *E. gracilis* is highly plastic and can be modulated by light conditions and strain background. For example, Sun et al. [32] observed spectrum-driven increases in paramylon productivity, with associated proteomic shifts in key carbon metabolism enzymes, such as fructokinase-1 and chloroplastic fructose-1,6-bisphosphatase. These enzymes likely overlap with hub genes in our trait-associated modules, offering experimentally testable targets for strain optimization. However, our transcriptomic data revealed only limited changes in paramylon pathway genes, pointing to post-transcriptional control. This aligns with proteomic evidence that the paramylon synthase EgGSL1 is detected at the protein level only under phototrophic conditions despite constitutive transcription [63]. Post-translational control of paramylon-degrading enzymes may further tune net accumulation [64]. Consequently, the observed spectrum-dependent paramylon yields are likely mediated by protein abundance and enzyme activity rather than by transcript levels.

Lipid accumulation under different spectra appears to be driven by two overlapping mechanisms. First, light quality affects the redox and ATP balance via photosystem operation, influencing lipid biosynthesis capacity. Second, carotenoid and lipid droplet accumulation may be co-regulated under light-induced stress conditions. Kato et al. [27] demonstrated that high-light intensity increases both lipid globules and carotenoid content in *Euglena*, reinforcing this coupling.

Furthermore, the impact of trophic mode and carbon source on lipid composition in *E. gracilis* is well-documented [65]. Our results expand this understanding by associating light-driven transcriptional modules with lipid accumulation trends, indicating that specific gene networks may channel excess reductants into lipid biosynthesis under defined spectral regimes.

Overall, the integration of physiological and transcriptomic data in this study provides new insights into how light quality governs carbon flux partitioning in *E. gracilis*, and offers candidate targets for engineering light-responsive pathways to enhance the yield of paramylon (as a functional bioproduct) or lipids (for biofuel and bioproduct applications).

### Photoreceptor signaling as an upstream layer of spectrum-responsive gene modules

Light in *Euglena gracilis* serves not only as an energy source but also as a spectral signal transduced via specific photoreceptors. This dual role supports the emergence of spectrum-segregated gene co-expression modules observed in our WGCNA. The presence of rhodopsin-like photoreceptors [18] and flavin-associated photoreception mechanisms localized to the flagellar apparatus [20] provides a molecular basis for blue and red light-specific signaling. These systems have the potential to influence nuclear–plastid communication, pigment biosynthesis, cytoskeletal regulation, and broader acclimation responses. This dual role of blue light provides a mechanistic basis for the coordinated accumulation of carotenoids and paramylon observed in our co-expression modules, triggering transcriptional programs that enhance both photoprotection and carbon storage [26].

*Euglena*’s ability to perform light-dependent vertical migration introduces another axis of regulation. High radiation can initiate behavioral repositioning, which in turn alters photosynthetic efficiency within vertical gradients [28]. These behaviors are cytoskeleton-dependent and may explain why some white-light-associated modules in our network were enriched for cytoskeletal and motility genes. Altogether, the observed WGCNA module separation reflects upstream photoreceptor signaling cascades operating in a spectrum-dependent manner.

### Implications for spectral engineering in bioproduct-focused cultivation

From a bioprocessing standpoint, these findings support the strategic use of light spectra to influence carbon allocation in *E. gracilis*. However, this form of control is conditional on adequate inorganic carbon supply. Our results, together with previous studies, suggest a two-step optimization framework: first, CO_2_ levels must be sufficient to stabilize photosynthetic performance and avoid stress-induced productivity losses; second, the light spectrum can then be tuned to bias biosynthetic pathways toward desired products. Consistent with prior studies [30, 31], our WGCNA-derived carotenoid- and paramylon-associated modules provide molecular entry points for tuning carbon partitioning, contingent on adequate CO₂ supply.

In practice, cultivation strategies could implement phased spectral regimens: an initial phase prioritizing growth under spectra that maximize *Fv*/*Fm*, followed by a shift to product-enriching spectra (e.g., blue-dominant conditions) once biomass is established. Such regimens may enhance yields of carotenoids or paramylon without compromising overall productivity.

### Trait-resolved networks enable targeted engineering of spectrum responses

A key advantage of WGCNA in this context is its ability to reduce transcriptome complexity while preserving biological interpretability. Modules associated with *Fv*/*Fm*, pigment accumulation, paramylon, and protein content displayed strong and statistically significant trait correlations, indicating that these networks capture meaningful physiological variation.

The identified modules align with known pathways: the carotenoid-associated network is enriched for isoprenoid biosynthesis genes, such as *EgcrtB* [27], and the paramylon-associated module contains glycolytic/gluconeogenic regulators, such as fructokinase and *FBPase* [32], which may correspond to hub genes identified in the relevant co-expression modules.

These modules offer a prioritized list of candidates for further investigation. Experimental validation using qRT-PCR, enzymatic assays, and functional perturbation (e.g., gene knockdown or overexpression) could establish causal relationships between hub genes and product accumulation, moving from correlative networks to mechanistic understanding.

### Limitations and future directions

While our findings highlight robust module–trait relationships, several limitations warrant discussion. First, *Euglena* exhibits complex responses that integrate metabolic and behavioral regulation. Some modules may reflect the transcriptomic output of both plastidic and motility programs, particularly under broad-spectrum light that affects both photosynthesis and cell behavior. Decoupling these components—for instance, through use of non-motile mutants or controlled mixing—could clarify the underlying contributions.

Second, time-resolved transcriptomics would be valuable. Previous studies demonstrate that blue light induces acclimatory changes over time, such as pigment remodeling and tolerance acquisition [26]. Capturing transcriptomic snapshots at early, mid, and late phases would help resolve the dynamics of signaling vs. metabolic modules.

Third, while total lipid content was quantified, more detailed analysis of lipid species and fatty acid composition is needed. Historical work shows that light, carbon source, and trophic mode all influence lipid profiles in *Euglena* [63], and future work should incorporate targeted lipidomics to better link transcript modules to specific lipid biosynthetic pathways.

While the WGCNA approach has identified highly connected hub genes within the carotenoid- and carbon-associated modules, the present study is intended as a hypothesis-generating investigation rather than a mechanistic conclusion. The correlations reported here should be viewed as a resource for prioritizing candidates for future validation. As a logical next step, the expression patterns of key hub genes, such as [gene name(s) if available], will be verified by qPCR under the same light conditions, and targeted gene manipulation studies will be required to test their regulatory roles directly. We believe this transparent discussion of limitations does not diminish the value of the network-level insights but instead provides a clear roadmap for follow-up experiments.

## Conclusions

This study demonstrates that light-quality functions as a tunable parameter to modulate both growth and bioproduct synthesis in *Euglena gracilis*. Both blue and red light enhanced biomass relative to white light, with carbon dioxide enrichment further supporting higher cell densities across all spectral conditions. Blue light specifically promoted the accumulation of chlorophyll a, chlorophyll b, and total carotenoids, alongside a marked increase in the storage polysaccharide paramylon. In contrast, red light, particularly when combined with elevated carbon dioxide, led to maximal lipid accumulation. Morphological changes under blue and red light—shifts from elongated to spindle or spherical forms—were consistent with underlying cytoskeletal remodeling detected at the transcriptome level.

At a system level, weighted gene co-expression network analysis (WGCNA) resolved the transcriptome into twelve discrete modules, each showing defined relationships with physiological traits and treatments. Notably, a turquoise module enriched in photosystem and photoprotection genes correlated with photochemical efficiency (*Fv*/*Fm*); a green module tracked total carotenoids; and a red module captured the coordinated effect of blue light on chlorophyll, carotenoids, and paramylon. Other modules, including brown and black, associated with protein content and white-light exposure, respectively, pointing to spectrum-dependent modulation of translation, cell structure, and transport pathways. These module–trait linkages aligned with differentially expressed genes involved in photosynthesis and photoreception, providing a mechanistic framework connecting spectral input to carbon allocation and plastid function.

From an applied perspective, these findings support targeted use of light spectra to guide bioproduct yields: blue-dominant conditions favor carotenoid and paramylon production while preserving photochemical function; red-dominant conditions under high CO₂ favor lipid accumulation; and staged lighting strategies may combine these outcomes over the cultivation cycle. Module eigengenes serve as compact biomarkers for process monitoring, while high-connectivity genes within trait-associated modules represent promising targets for future validation and engineering efforts.

This study is not without limitations. Incomplete gene annotation, limited temporal resolution, and the absence of direct functional assays restrict causal interpretation. Future research should include time-resolved transcriptomics, targeted gene perturbation, and detailed metabolite flux analyses. Factorial designs exploring spectral distribution, intensity, and carbon supply will further refine optimal cultivation strategies.

In summary, this work provides an integrative framework linking light spectrum to transcriptomic architecture and bioproduct formation in *Euglena gracilis*. These findings contribute to the rational design of light-driven strategies for microalgal biorefineries focused on sustainable production of carotenoids, paramylon, and lipids.

## Supplementary Information

Additional file1 (DOCX 1978 KB)

## Data Availability

The data for this study have been deposited in the European Nucleotide Archive (ENA) at EMBL–EBI under accession number PRJEB105942. The datasets used and/or analyzed during the current study are available from the corresponding author on reasonable request.
