# Supplementary material for: Light quality rewires module–trait networks in Euglena gracilis to drive carotenoid and paramylon accumulation
Source: Biotechnol Biofuels Bioprod. 2026 May 29;19:57. doi: 10.1186/s13068-026-02774-2 (PMC13422297; doi:10.1186/s13068-026-02774-2)

**Supplementary Information**

Manuscript title (placeholder): Light quality rewires module–trait networks in Euglena gracilis to drive carotenoid and paramylon accumulation

This supplementary document accompanies the main manuscript and contains additional methods, figures, and tables (S1–S10).

**Supplementary Methods**

**S1. Additional details for WGCNA**

We constructed a signed-hybrid weighted gene co-expression network using variance-stabilized counts. Outlier detection used hierarchical clustering and the goodSamplesGenes routine; outliers were excluded where necessary. Soft-thresholding power (β) was selected with pickSoftThreshold to approach scale-free topology; β = 30 was used. Topological overlap (TOM) was computed; genes were hierarchically clustered by TOM dissimilarity. Modules were detected by Dynamic Tree Cut and merged at mergeCutHeight = 0.25, yielding 12 color-labeled modules. Module eigengenes (MEs) were correlated (Pearson) with traits (Blue/Red/White dummies; Fv/Fm; chlorophyll a/b; total carotenoids; paramylon; total lipids; protein; morphology index). P values used the Student t approximation with Benjamini–Hochberg false discovery rate (FDR) control. Gene significance (GS) and module membership (kME) were computed to nominate high-GS/high-kME hub candidates.

**S2. Export to Cytoscape for module subnetworks**

For each trait-associated module, TOM edges above a threshold (e.g., top 5–10% by TOM) were exported as a 3-column edge list (source, target, weight) and imported into Cytoscape. Nodes were sized by kME and colored by GS for the relevant trait. This visualization emphasizes intramodular connectivity and the placement of candidate hub genes.

**Supplementary Figures**

**Figure S1. The spectra of different color LEDs. A-D, red LED (****635 nm), Blue LED (453 nm), White LED (450-760 nm), and Natural light, respectively.**

**
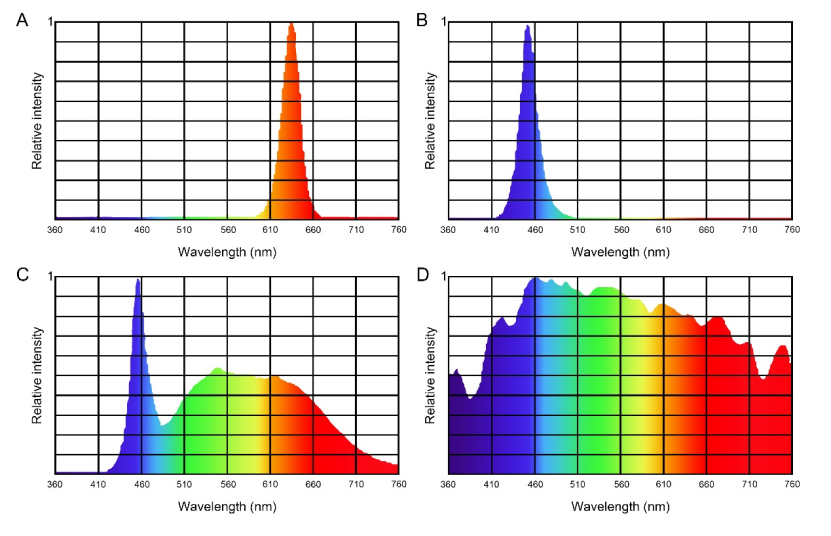
**

**Figure S2. Sample clustering with trait color bars (WGCNA QC).**


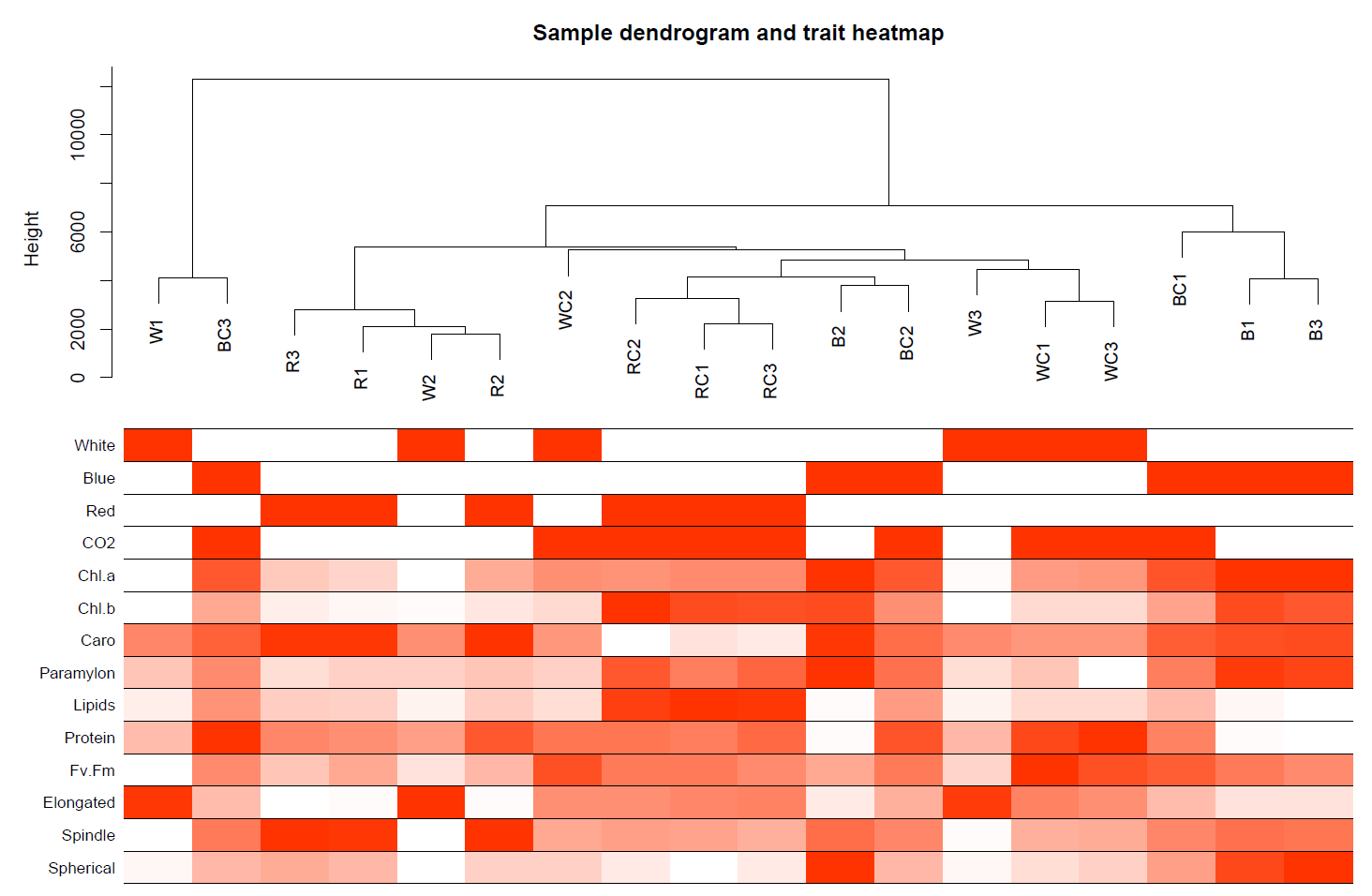


**Figure S3. Soft-threshold selection for network construction.**


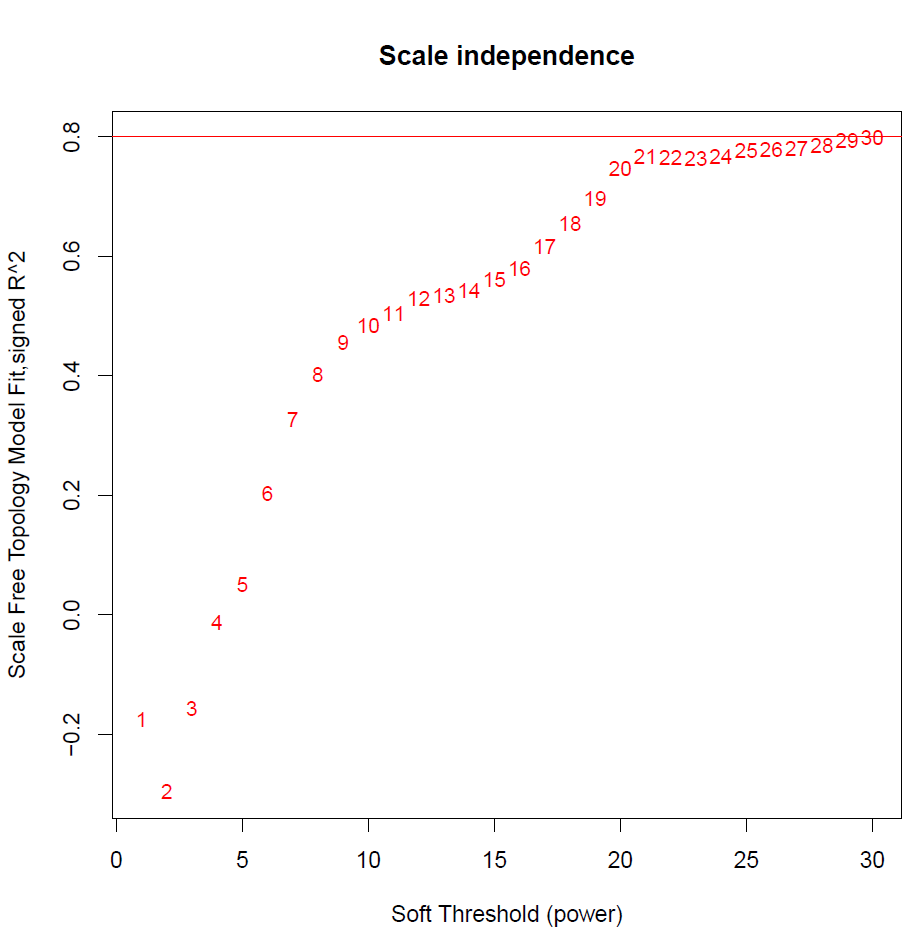

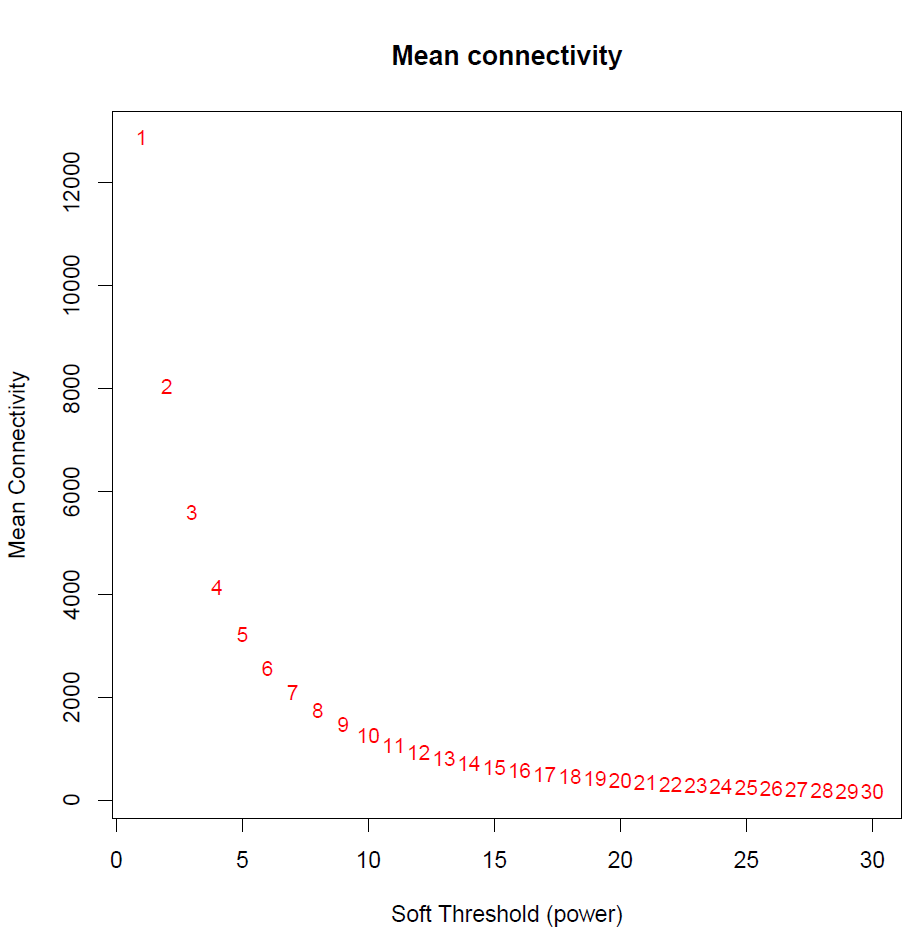


**Figure S4. Gene dendrogram and module assignment.**


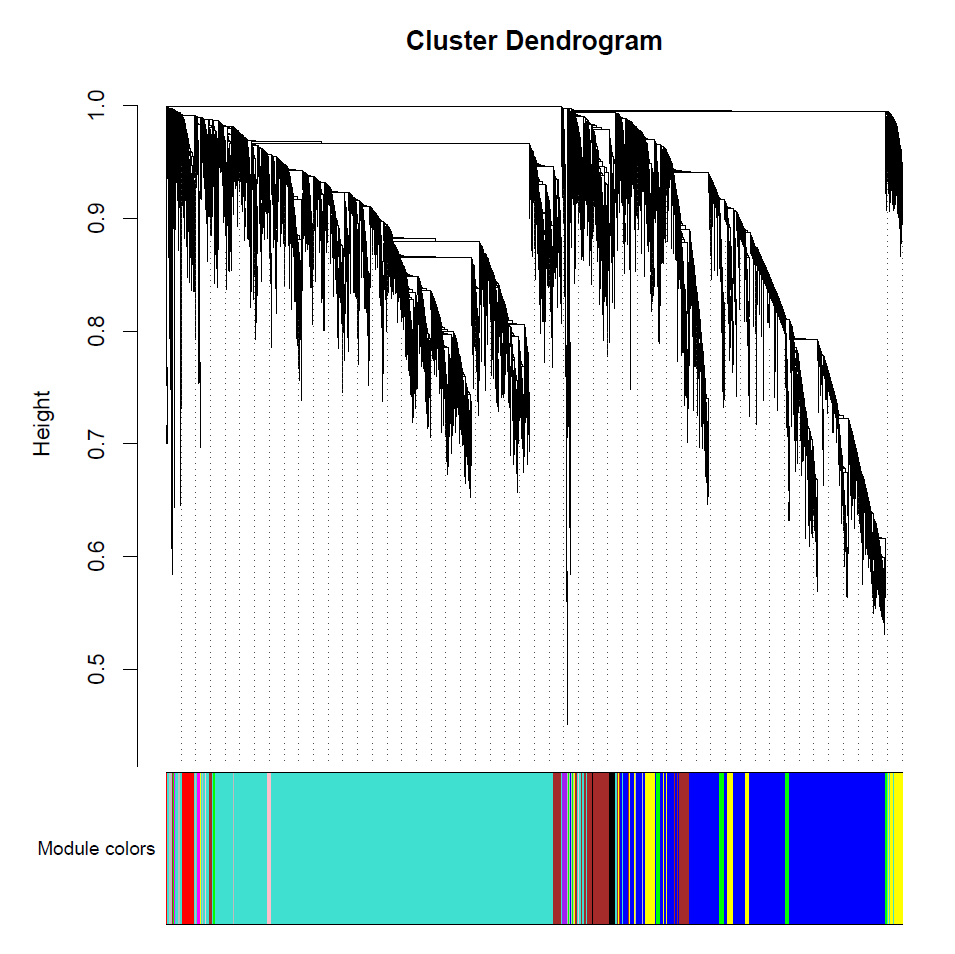

Supplement: Supplementary file 1 — Additional file1 (DOCX 1978 KB) [file 13068_2026_2774_MOESM1_ESM.docx]
